# Success and complications by team composition for prehospital pediatric intubation: children also need physicians!

**DOI:** 10.1186/s13054-020-03029-8

**Published:** 2020-06-15

**Authors:** Romain Jouffroy, Stéphanie Fogel, Peter Jones, Benoît Vivien

**Affiliations:** SAMU de Paris, SMUR Pédiatrique Necker, Service d’Anesthésie-Réanimation, Centre Hospitalier Universitaire Necker - Enfants Malades, AP-HP.Centre, and Université de Paris, Paris, France

To the Editor,

Garner et al. [1] reported that physician teams had higher rates of pediatric intubation success and lower rates of overall airway complications than other team types and that prehospital teams including physicians should be mobilized whenever practicable for critically ill children requiring prehospital intubation.

Undoubtedly, the authors should be congratulated for this thorough and systematic review and meta-analysis in the area of prehospital airway management, a question of the utmost importance especially for children. Nevertheless, we consider that their interpretation requires some caution.

As stated by the authors, pediatric intubation is infrequent in the prehospital setting [2, 3]. As a rare event, pediatric intubation requires personal experience and a minimum number of acts per year, in order to limit complications. Small numbers of children included in clinical studies also make extrapolation to the general pediatric population difficult. First, we are surprised by the rate of endobronchial intubation (3%, 95% CI [0–11]) in the physician teams. Second, due to the absence of international recommendations, practices between countries are heterogeneous, which make comparisons difficult. The results are influenced by the geographical origin of the studies; for instance, 65% of studies come from the USA where the prehospital emergency services are based on paramedics. Third, from a methodological point of view, we wonder why the authors did not consider an alternative group of physicians who do not use muscle relaxants. Indeed, emergency services traditionally intubate children without muscle relaxants [1], and the results of Garner et al. strongly suggest an effect of muscle relaxants on outcomes, whoever the operator. Fourth, the wide age range used for pediatric definition possibly influences the results because airway access conditions vary greatly between infancy and adolescence. From the age of 15 years, airway access conditions are similar to those of an adult. Last but not least, it is surprising to see six studies with muscle relaxant administration for cardiac arrest. In this situation, the vocal cords are open and intubating conditions are usually considered as optimal.

Beyond these limitations, we fully agree with Garner et al. that airway management is a critical component of prehospital care, particularly in children who do not tolerate hypoxemia well. To achieve this goal, we believe that the presence of an experienced physician in the prehospital setting is essential. Despite these limitations, the study of Garner et al. reinforces the need for a physician’s presence in prehospital emergency response systems to improve pediatric patients’ outcome.

## Data Availability

Not applicable
